# Impact of Angiotensin‐Converting Enzyme Inhibitor on Coronary Artery Calcification Evaluated by Intravascular Ultrasound: A Retrospective Cohort Study

**DOI:** 10.1002/hsr2.70900

**Published:** 2025-06-11

**Authors:** Daisuke Kanda, Akihiro Tokushige, Kenta Ohmure, Daichi Fukumoto, Hirokazu Shimono, Hiroyuki Tabata, Nobuhiro Ito, Takuro Kubozono, Mitsuru Ohishi

**Affiliations:** ^1^ Department of Cardiovascular Medicine and Hypertension, Graduate School of Medical and Dental Sciences Kagoshima University Kagoshima Japan

**Keywords:** angiotensin‐converting enzyme inhibitor, calcified nodule, coronary calcification, intravascular ultrasound, percutaneous coronary intervention

## Abstract

**Background and Aims:**

Coronary artery calcification (CAC) is a predictor of poor prognosis after percutaneous coronary intervention (PCI), and its treatment depends on calcification severity assessed by intravascular imaging such as intravascular ultrasound (IVUS). We aimed to investigate the factors associated with CAC severity and clinical outcomes, with a particular focus on the potential impact of angiotensin‐converting enzyme inhibitor (ACEI) use.

**Methods:**

We retrospectively analyzed 630 patients with stable coronary artery disease who underwent IVUS‐guided PCI between January 2018 and March 2023. Calcification severity was assessed using IVUS, and patients were grouped into moderate/severe and none/mild calcification. Outcomes included all‐cause death and major adverse cardiovascular and cerebrovascular events (MACCEs).

**Results:**

Patients with moderate/severe calcification had a significantly lower survival rate and a higher incidence of MACCEs (*p* = 0.02 and *p* < 0.001, respectively). Similarly, patients with calcified nodules had worse outcomes (*p* = 0.005 and *p* < 0.001, respectively). ACEI use was independently associated with reduced odds of moderate/severe calcification [OR: 0.56, 95% confidence intervals (CI): 0.36–0.90, *p* = 0.02] and calcified nodules (OR: 0.10, 95% CI: 0.01–0.74, *p* = 0.02). In patients with moderate/severe calcification, ACEI use was associated with a lower MACCE rate (*p* = 0.04).

**Conclusion:**

IVUS‐assessed moderate/severe calcification is a strong predictor of all‐cause death and MACCE in patients with CAD undergoing PCI. ACEI use was associated with less severe calcification and a lower incidence of MACCE in these patients. Evaluation of calcification may help identify high‐risk patients and guide anti‐calcification treatment strategies.

## 1 Introduction

Vascular calcification is a pathological vascular disorder associated with various diseases, including atherosclerosis, hypertension, aortic valve stenosis, coronary artery disease (CAD), diabetes, and chronic kidney disease [1]. It is recognized not only as a marker of advanced atherosclerosis but also as an active and regulated process contributing to plaque instability and adverse cardiovascular outcomes. Vascular calcification is a poor prognostic factor because it occurs locally in the coronary arteries and arteries throughout the body.

Coronary artery calcification (CAC), characterized by calcium deposition in the coronary arterial walls due to advanced atherosclerosis, is highly prevalent in patients with CAD and is observed in over 90% of men and 67% of women aged > 70 years [2].

CAC is not only a marker of advanced atherosclerosis but also a strong and independent predictor of future cardiovascular events. Large‐scale cohort studies have demonstrated that CAC provides incremental prognostic value beyond traditional risk factors, including hypertension, diabetes, and dyslipidemia [3]. Because CAC reflects the total atherosclerotic burden, it has been widely used for cardiovascular risk stratification, even in asymptomatic individuals, and has been shown to predict cardiovascular events more accurately than other conventional risk factors [4, 5, 6].

Although CAC cannot be reversed, its progression can be slowed by lifestyle interventions and medical therapy, including smoking cessation, blood pressure and lipid control, weight management, and pharmacological therapies targeting atherosclerosis [7]. Nonetheless, CAC is significantly associated with major adverse cardiac events in patients with CAD [2], and contributes to poor outcomes after percutaneous coronary intervention (PCI) and procedural complications such as vessel dissection, perforation, and stent complications [8, 9].

CAC can be detected using various imaging modalities [10]. The coronary artery calcium score assessed by computed tomography (CT) is widely used [11], while intravascular ultrasound (IVUS) has demonstrated superior detection of coronary calcification compared to conventional angiography [12].

Current treatment strategies for calcified lesions during PCI, including rotational atherectomy (RA), orbital atherectomy system (OAS), intravascular lithotripsy, and scoring balloon angioplasty, are determined based on calcium severity observed on intravascular imaging [12, 13].

Despite advances in the detection and management of CAC, vascular calcification is caused by a complex interplay of many factors and remains a significant challenge in clinical practice. Recent studies have revealed that vascular calcification is associated with cellular and molecular mechanisms such as chronic inflammation, mitochondrial dysfunction, and programmed cell death [14, 15, 16].

Agiotensin‐converting enzyme inhibitors (ACEIs) have been shown to reduce morbidity and mortality in patients with cardiovascular diseases, including heart failure and left ventricular systolic dysfunction [17]. A systematic review of randomized trials has also demonstrated that ACEIs reduce all‐cause mortality and cardiovascular events in patients with atherosclerosis [18]. Several studies suggest that ACEIs exert vasculoprotective effects beyond their antihypertensive effects by reducing oxidative stress and inflammation and may inhibit vascular smooth muscle cell calcification [19]. These findings suggest a potential mechanistic link between ACEI therapy and modulation of vascular calcification.

However, the relationship between ACEI use and the severity of coronary calcification—particularly as assessed by IVUS in patients with stable CAD undergoing PCI—remains unclear. Previous studies using IVUS have reported factors associated with poor stent expansion in calcified lesions [13, 20, 21], but the clinical predictors of IVUS‐assessed calcification severity itself have not been adequately investigated.

Therefore, the aim of this study was to investigate clinical factors, including the use of ACEIs, associated with the severity of coronary calcification detected by IVUS and to examine their impact on clinical outcomes in patients with stable CAD undergoing PCI.

## 2 Methods

### 2.1 Ethical Considerations

This study protocol was reviewed and approved by the Epidemiological Research Ethics Committee of Kagoshima University Hospital (approval number: 200144). Written informed consent was obtained from all participants before enrollment, either at the time of hospital admission or during outpatient visits, after a detailed explanation of the study objectives, risks, and confidentiality procedures. This study adhered to the principles of the Declaration of Helsinki (1975) and its later amendments. The patient data were anonymized and stored securely to ensure confidentiality.

### 2.2 Study Population

The inclusion criteria were as follows: (1) patients with stable coronary artery disease who underwent elective PCI at our institution between January 2018 and March 2023, (2) successful IVUS‐guided PCI with a second‐generation drug‐eluting stent (DES) and/or drug‐coated balloon (DCB), and (3) availability of IVUS data before stent implantation. Exclusion criteria were as follows: (1) presentation with acute coronary syndrome, (2) inability to perform IVUS due to severe vessel tortuosity or technical limitations, and (3) follow‐up duration of less than 6 months. Among the 677 screened patients, 47 were excluded (45 due to IVUS inaccessibility and 2 due to early loss to follow‐up), resulting in a final cohort of 630 patients.

Myocardial ischemia in patients with stable CAD was evaluated using the fractional flow reserve (FFR) measured during invasive coronary angiography, myocardial perfusion single‐photon emission CT, or FFR derived from coronary CT angiography. All patients were administered statins before and/or after PCI for secondary prevention of CAD.

### 2.3 Measurements, Including Laboratory Values and Definitions

Blood samples were collected before PCI. Serum concentrations of albumin, high‐density lipoprotein cholesterol, low‐density lipoprotein cholesterol (LDL‐C), and fasting plasma glucose were measured after a 12‐h fast. Body mass index (BMI) was calculated as body weight in kilograms divided by height in meters squared (kg/m^2^). The Japan Society for the Study of Obesity stipulates that the ideal BMI for the Japanese population is 22 kg/m^2^, and obesity is defined as a BMI of 25 kg/m^2^ [20]. Therefore, we used 22 kg/m^2^ as the ideal BMI. Patients were classified as current smokers if they had a history of smoking at least 100 cigarettes in their lifetime and had continued to smoke at the time of hospital admission. Follow‐up assessments were conducted either at our institution or by the patients' primary care physicians.

### 2.4 PCI Procedure and Assessments of Calcification in Culprit Lesions

PCI was performed using the standard technique via a transfemoral or transradial approach. The surgeon decided whether the final device should be a second DES, DCB, or a combination of both. IVUS imaging was performed using a 60‐MHz mechanical IVUS catheter (Altaview; Terumo, Tokyo, Japan) that automatically recorded at a pullback rate of 3 mm/s or 9 mm/s (30 or 10 frames/s), inserted over a 0.014‐inch guidewire before lesion modification using a balloon, and before and after stent implantation. The images were digitally stored and analyzed offline by interventional cardiologists. Calcification severity was defined based on IVUS data. Moderate/severe calcification in the target lesion was defined as calcifications having both calcium arch > 180° mm and calcium length > 5 mm on IVUS [21]. Other findings were defined as none/mild calcification. Calcified nodules were defined as those having a convex shape on the luminal surface and a dense, irregular surface appearance on IVUS images [22, 23]. Target lesions with calcified nodules were included in the moderate/severe calcification group. In particular, all severely calcified lesions required some lesion modification with scoring balloons (cutting balloon, non‐slip element, and scoreflex), RA (Boston Scientific, Natick, MA, USA), OAS (Cardiovascular Systems Inc. Diamondback 360, St. Paul, MN, USA), and/or their combinations [24]. For lesion modification of severely calcified lesions, the choice of device was based on the surgeon's discretion, accounting for the lesion, IVUS findings, and patient background.

### 2.5 Clinical Outcomes

Clinical outcomes were retrospectively examined during the follow‐up period. Follow‐up data were obtained from medical records, from the primary physician, or via telephone interviews with the patient or relatives. The primary outcome measure was the cumulative incidence of all‐cause death. The secondary outcome measures were the cumulative incidence rates of all‐cause death and MACCEs. MACCEs included all‐cause death, nonfatal myocardial infarction, and ischemic stroke. The patients were categorized into moderate/severe calcification and none/mild calcification groups. Additionally, we compared the relationship of baseline patient characteristics between the two groups categorized according to the degree of calcification of the culprit lesion with MACCE, including all‐cause death.

### 2.6 Statistical Analysis

For descriptive statistics, categorical variables are presented as frequencies and percentages. The normality of continuous variables was assessed using the Shapiro–Wilk test. Normally distributed data are expressed as the mean ± standard deviation, and non‐normally distributed data are shown as medians with interquartile ranges. Categorical variables were analyzed using Fisher's exact test for between‐group comparisons. Continuous variables were compared between the moderate/severe calcification and none/mild calcification groups using the Student's *t*‐test for normally distributed values or Wilcoxon's rank‐sum test for non‐normally distributed values. Time‐to‐event outcomes, including all‐cause death and MACCE, were analyzed using the Kaplan–Meier method. Log‐rank tests were used to compare survival curves between the following groups: moderate/severe versus none/mild calcification, presence versus absence of calcified nodule lesions, and ACEI use versus non‐use. To adjust for potential confounding variables and assess the independent effect of factors on time‐to‐event outcomes, Cox proportional hazards regression analysis was performed. The results were reported as hazard ratios (HRs) with corresponding 95% confidence intervals (CIs). The proportional hazard assumptions were tested using Schoenfeld residuals. Logistic regression analysis was conducted to calculate the odds ratios (ORs) for the presence of moderate/severe calcifications and calcified nodules. Multivariable models included clinically relevant covariates based on the prior literature and baseline imbalances. An interaction term between chronic kidney disease (CKD) and ACEI use was included in the regression models to explore potential effect modification, given the known renoprotective properties of ACEI and the strong association between CKD and vascular calcification. Cutoff values for LDL‐C, serum albumin, and hs‐CRP were determined based on established clinical guidelines and previous reports showing their prognostic relevance in coronary artery disease (CAD). Specifically, LDL‐C < 70 mg/dL was selected according to Japanese secondary prevention guidelines [25], and thresholds for albumin and hs‐CRP were selected based on the literature indicating their association with adverse cardiovascular outcomes [26, 27]. All statistical analyses were performed using the JMP version 17.0 (SAS Institute Inc., Cary, NC, USA). Statistical significance was set at a two‐tailed *p* value < 0.05.

## 3 Results

### 3.1 Baseline Characteristics

Table 1 presents the baseline clinical characteristics of the patients. Their median age was 69 years (range, 63–76 years), and most patients were male (74%). The mean/median BMI of the patients was 23.5 (range, 21.3–26.1) kg/m^2^. More than half of the patients had the following risk factors: hypertension, 86%; current smokers, 20%; dyslipidemia, 75%; diabetes mellitus, 60%; and CKD, 56%. Regarding medication use, most patients were on statins (80%), approximately half were on calcium channel blockers (50%) and ARBs (47%), while fewer were on β‐blockers (39%) and ACEIs (17%).

**Table 1:** Patient characteristics.

Variables	Overall	Moderate/severe calcification group	None/mild calcification group	*p* value
	(*n* = 630)	(*n* = 254)	(*n* = 376)	
Age, years	69 [63,76]	71 [64,78]	68 [60,74]	0.001
Sex (male), *n* (%)	467 (74)	190 (75)	277 (74)	0.54
BMI, kg/m^2^	23.5 [21.3, 26.1]	22.8 [20.9, 25.3]	23.8 [21.8, 26.6]	0.78
Risk factors, *n* (%)				
Hypertension	541 (86)	224 (88)	317 (84)	0.19
Diabetes mellitus	378 (60)	160 (63)	218 (58)	0.21
Dyslipidemia	475 (75)	185 (73)	290 (77)	0.22
Current smoking	125 (20)	41 (16)	84 (22)	0.06
CKD	355 (56)	176 (69)	179 (48)	< 0.001
Medication, *n* (%)				
Calcium channel blocker	313 (50)	126 (50)	187 (50)	> 0.99
ACEI	110 (17)	34 (13)	76 (20)	0.03
ARB	294 (47)	124 (49)	170 (45)	0.41
β‐Blocker	243 (39)	116 (46)	127 (34)	0.004
Statin	505 (80)	208 (82)	297 (79)	0.41
Laboratory data				
hs‐CRP, mg/dL	0.14 [0.05, 0.39]	0.15 [0.05, 0.37]	0.14 [0.05, 0.40]	0.43
LDL‐C, mg/dL	71 [58,95]	70 [55,90]	74 [61,98]	0.001
HDL‐C, mg/dL	49 [41,58]	50 [42,59]	48 [40,57]	0.11
Albumin, g/dL	4.0 [3.6, 4.3]	3.9 [3.6, 4.3]	4.1 [3.7, 4.3]	< 0.001
FPG, mg/dL	110 [9, 131]	109 [92, 130]	111 [96, 131]	0.12

*Note:* Values are shown as *n* (%), mean ± standard deviation, or median with interquartile range.

Abbreviations: ACEI, angiotensin‐converting enzyme inhibitor; ARB, angiotensin II receptor blocker; BMI, body mass index; CKD, chronic kidney disease; FPG, fasting plasma glucose; HDL‐C, high‐density lipoprotein cholesterol; hs‐CRP, high‐sensitivity C‐reactive protein; LDL‐C, low‐density lipoprotein cholesterol.

Moderate/severe calcification, assessed using IVUS, was observed in the culprit lesions of 254 (40%) patients. The median age was significantly higher in the moderate/severe calcification group than in the none/mild calcification group (71 years [range, 64–78 years] vs. 68 years [range, 60–74 years], *p* < 0.001). The frequency of CKD (69% vs. 48%, *p* < 0.001) and β‐blocker use (46% vs. 34%, *p* = 0.004) was significantly higher in the moderate/severe calcification group than in the none/mild calcification group. The rate of ACEI use was statistically significantly lower in the moderate/severe calcification group than in the none/mild calcification group (13% vs. 20%, *p* = 0.03). Serum LDL‐C and albumin levels were significantly lower in the moderate/severe calcification group than in the noncalcification group (*p *= 0.001 and *p* < 0.001, respectively) (Table 1). There were no significant differences between the groups in terms of sex, BMI, other cardiovascular risk factors, other medications, or other laboratory data.

### 3.2 Clinical Outcomes After PCI

The median follow‐up duration was 844 days (interquartile range, 403–1363 days). Overall, 47 (7%) patients died, and MACCEs, including all‐cause death occurred in 100 (16%) patients. The incidences of all‐cause death (11% vs. 5%, *p* = 0.02) and MACCEs (25% vs. 10%, *p* < 0.001) were statistically significantly higher in the moderate/severe calcification group than in the noncalcification group. There was no significant between‐group difference in the rates of nonfatal myocardial infarction and ischemic stroke; however, the revascularization rate was statistically significantly higher in the moderate/severe calcification group (13% vs. 3%, *p* < 0.001) (Table 2).

**Table 2:** All‐cause death and MACCE.

	Overall (*n* = 630)	Moderate/severe calcification group (*n* = 254)	None/mild calcification group (*n* = 376)	*p* value
All‐cause death	47 (7)	27 (11)	20 (5)	0.02
MACCE	100 (16)	63 (25)	37 (10)	< 0.001
Nonfatal myocardial infarction	5 (0.8)	2 (0.8)	3 (0.8)	> 0.99
Ischemic stroke	5 (0.8)	1 (0.4)	4 (1.1)	0.65
Revascularization	43 (7)	33 (13)	10 (3)	< 0.001

*Note:* Values are presented as *n* (%).

Abbreviation: MACCE, major adverse cardiovascular and cerebrovascular event.

**Figure 1:**
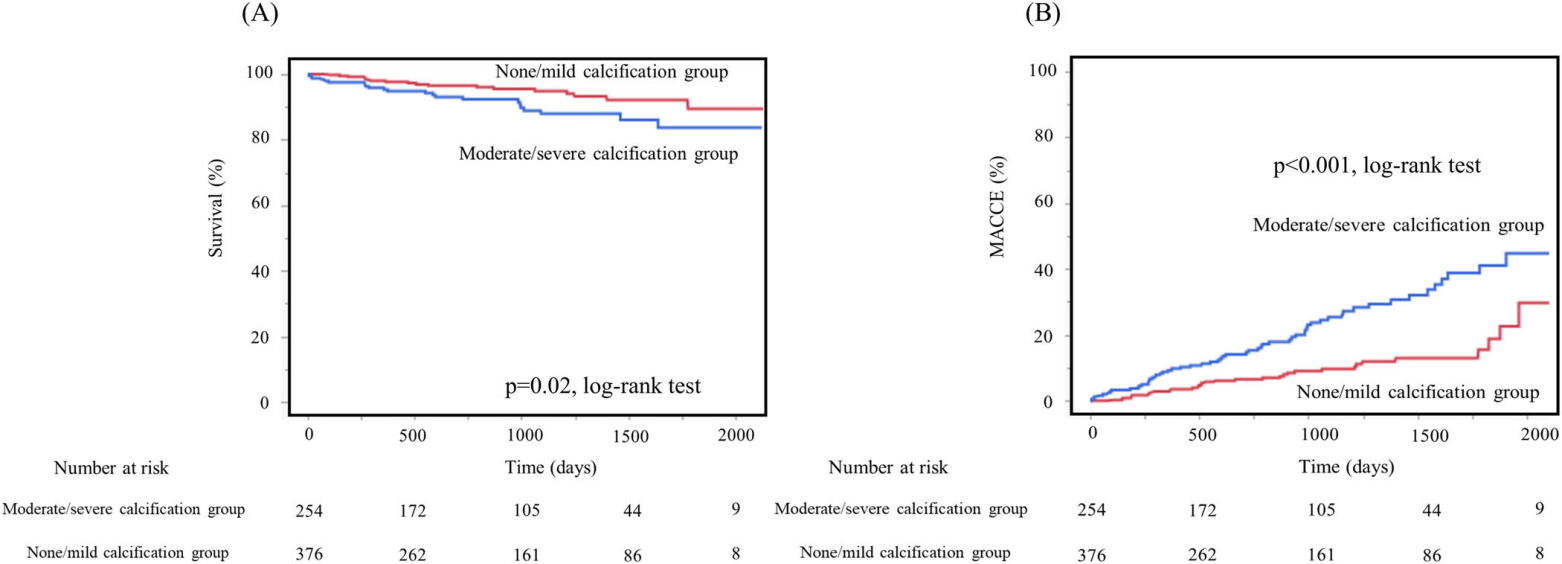
Comparison of Kaplan–Meier survival rates (A) and cumulative incidences of MACCEs (B) after PCI between the moderate/severe and none/mild calcification groups. Abbreviations: MACCEs, major cardiovascular and cerebrovascular events; PCI, percutaneous coronary intervention.

Kaplan–Meier analysis revealed that the moderate/severe calcification group had a statistically significantly lower survival rate (*p *= 0.02) and higher MACCE incidence (*p *< 0.001) than the none/mild calcification group (Figure 1A,B).

### 3.3 Factors Associated With Calcification

Age, CKD, ACEI, and β‐blocker use were significantly correlated with moderate/severe calcification. Multivariate logistic regression analysis revealed that age (OR: 1.03, 95% CI: 1.01–1.04, *p* = 0.002), CKD (OR: 2.15, 95% CI: 1.52–3.03, *p* < 0.001), and β‐blocker use (OR: 1.79, 95% CI: 1.26–2.52, *p* = 0.001) were positively associated with moderate/severe calcification. ACEI use (OR: 0.56, 95% CI: 0.36–0.90, *p* = 0.02) was inversely associated with moderate/severe calcification. The interaction *p* value between CKD and ACEI was 0.72 (Table 3).

**Table 3:** Logistic regression analysis of factors influencing moderate/severe calcification of the target lesion.

	Univariate analysis			Multivariate analysis		
	OR	(95% CI)	*p* value	OR	(95% CI)	*p* value
Age	1.03	(1.02–1.05)	< 0.001	1.03	(1.01–1.04)	0.002
Sex (male)	1.06	(0.74–1.52)	0.75			
BMI	0.96	(1.00–1.04)	0.06			
Hypertension	1.40	(0.87–2.23)	0.17			
Diabetes mellitus	1.23	(0.89–1.71)	0.20			
Dyslipidemia	0.80	(0.55–1.15)	0.22			
Current smoking	0.67	(0.44–1.01)	0.06			
CKD	2.48	(1.78–3.47)	< 0.001	2.15	(1.52–3.03)	< 0.001
Calcium channel blocker use	0.99	(0.72–1.37)	0.97			
ACEI use	0.41	(0.22–0.72)	0.003	0.56	(0.36–0.90)	0.02
ARB use	1.16	(0.84–1.59)	0.37			
β‐Blocker use	1.65	(1.19–2.28)	0.003	1.79	(1.26–2.52)	0.001
Statin use	1.20	(0.80–1.80)	0.37			
hs‐CR*p* ≥ 0.1 mg/dL	0.88	(0.64–1.22)	0.45			
LDL‐C > 70 mg/dL	0.75	(0.54–1.03)	0.07			
HDL‐C < 40 mg/dL	0.74	(0.49–1.10)	0.13			
Albumin < 3.5 g/dL	1.49	(0.96–2.33)	0.08			
FPG	0.998	(0.994–1.002)	0.39			

Abbreviations: ACEI, angiotensin‐converting enzyme inhibitor; ARB, angiotensin II receptor blocker; BMI, body mass index; CI, confidence interval; CKD, chronic kidney disease; FPG, fasting plasma glucose; HDL‐C, high‐density lipoprotein cholesterol; hs‐CRP, high‐sensitivity C‐reactive protein; LDL‐C, low‐density lipoprotein cholesterol; OR, odds ratio.

### 3.4 Prognostic Effect of the Presence of Calcified Nodules

**Figure 2:**
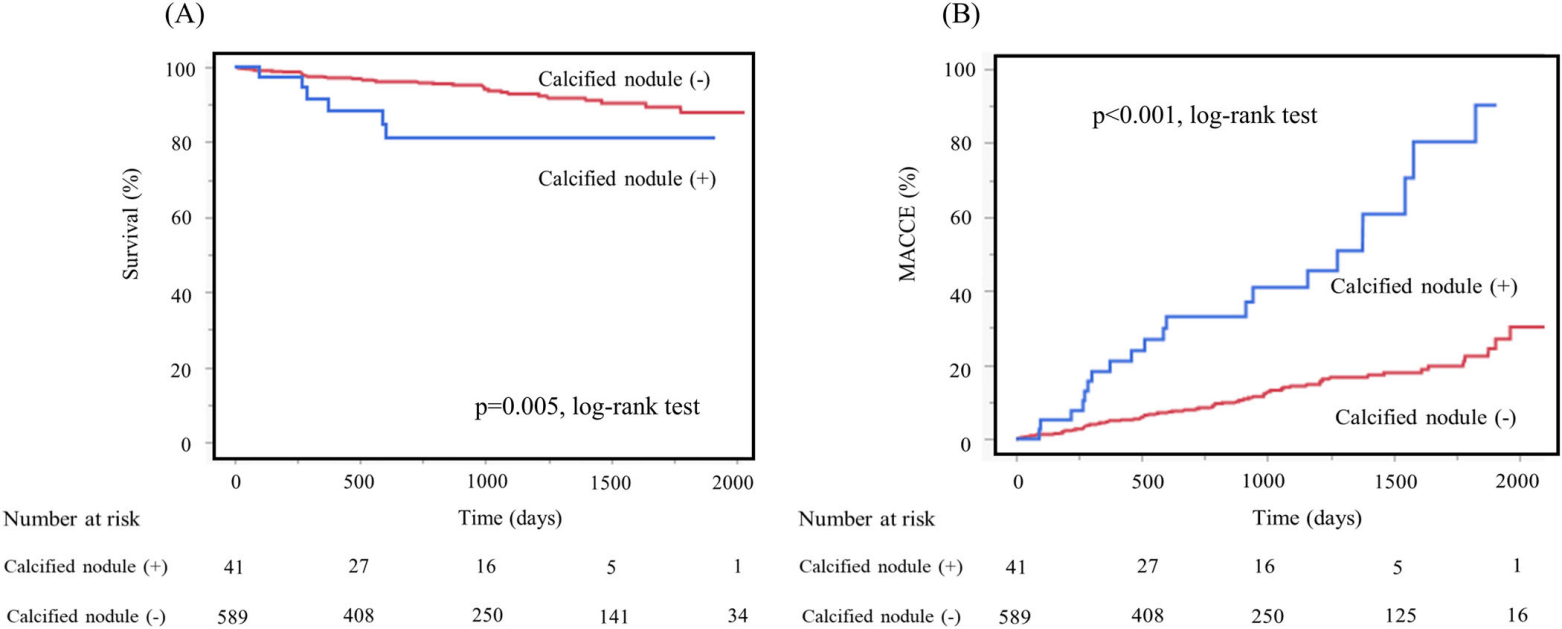
Kaplan–Meier analysis of survival rate (A) and cumulative incidences of MACCEs (B) after PCI in the groups with and without calcified nodule lesions. Abbreviations: MACCEs, major cardiovascular and cerebrovascular events; PCI, percutaneous coronary intervention.

Forty‐one patients in the moderate‐to‐severe calcification group had calcified nodules. Kaplan–Meier analysis showed that patients with calcified nodule lesions had a statistically significantly lower survival rate (*p *= 0.005) and significantly higher MACCE incidence (*p *< 0.001) than those without calcified nodule lesions (Figure 2A,B).

Multivariate logistic regression analysis revealed that CKD (OR: 3.29, 95% CI: 1.46–7.39, *p* = 0.004) and β‐blocker use (OR: 2.02, 95% CI: 1.06–3.88, *p* = 0.03) were positively associated with calcified nodules. ACEI use (OR: 0.10, 95% CI: 0.01–0.74, *p* = 0.02) was negatively associated with calcified nodules. The interaction *p* value between CKD and ACEI was 0.98 (Table 4).

**Table 4:** Logistic regression analysis of factors influencing calcified nodules in the target lesions.

	Univariate analysis			Multivariate analysis		
	OR	(95% CI)	*p* value	OR	(95% CI)	*p* value
Age	1.00	(0.97–1.03)	0.96	0.99	(0.96–1.02)	0.57
Sex (male)	2.12	(0.87–5.14)	0.09			
BMI	0.95	(0.87–1.03)	0.22			
Hypertension	7.03	(0.95–51.77)	0.06			
Diabetes mellitus	1.04	(0.55–2.00)	0.89			
Dyslipidemia	1.01	(0.48–2.12)	0.97			
Current smoking	0.68	(0.28–1.65)	0.39			
CKD	3.42	(1.55–7.53)	0.002	3.29	(1.46–7.39)	0.004
Calcium channel blocker use	1.32	(0.70–2.49)	0.98			
ACEI use	0.11	(0.01–0.81)	0.03	0.10	(0.01–0.74)	0.02
ARB use	1.67	(0.88–3.17)	0.12			
β‐Blocker use	1.93	(1.02–3.64)	0.04	2.02	(1.06–3.88)	0.03
Statin use	3.31	(1.004–10.00)	0.49			
hs‐CR*p* ≥ 0.1 mg/dL	1.41	(0.72–2.75)	0.31			
LDL‐C ≥ 70 mg/dL	0.69	(0.36–1.31)	0.26			
HDL‐C < 40 mg/dL	1.38	(0.67–2.84)	0.38			
Albumin < 3.5 g/dL	1.44	(0.64–3.23)	0.37			
FPG	0.997	(0.989–1.006)	0.55			

Abbreviations: ACEI, angiotensin‐converting enzyme inhibitor; ARB, angiotensin II receptor blocker; BMI, body mass index; CI, confidence interval; CKD, chronic kidney disease; FPG, fasting plasma glucose; HDL‐C, high‐density lipoprotein cholesterol; hs‐CRP, high‐sensitivity C‐reactive protein; LDL‐C, low‐density lipoprotein cholesterol; OR, odds ratio.

### 3.5 Effect of Post‐PCI ACEI on Calcified Lesion

**Figure 3:**
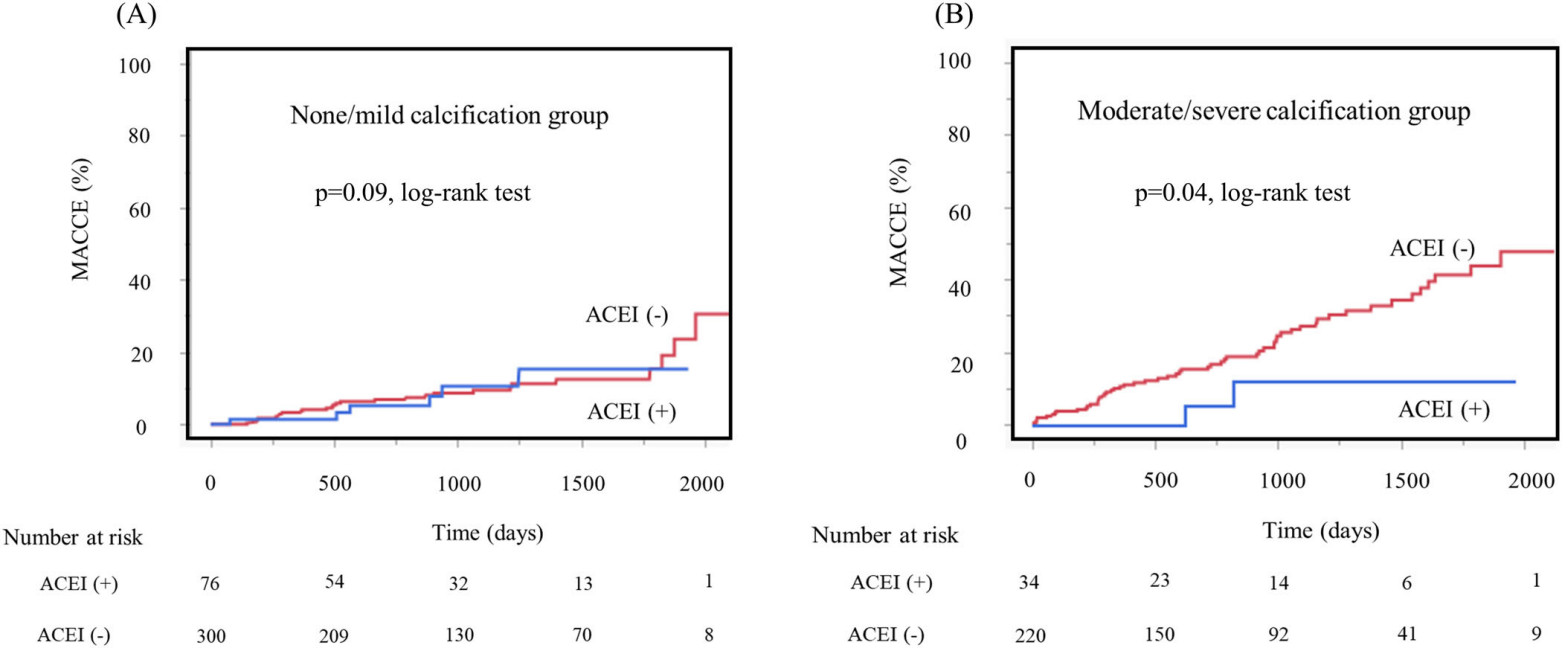
Kaplan–Meier analysis of cumulative incidences of MACCEs after PCI in the groups with and without ACEI use: (A) none/mild calcification group, (B) moderate/severe calcification group. ACEI, angiotensin‐converting enzyme inhibitor; MACCEs, major cardiovascular and cerebrovascular events; PCI, percutaneous coronary intervention.

Kaplan–Meier analysis showed that in the moderate/severe calcification group, the ACEI‐use group had a statistically significantly lower incidence of MACCEs than the non‐use ACEI group (*p *= 0.04). No significant difference was observed in the none/mild calcification group (*p *= 0.90) (Figure 3).

### 3.6 Kaplan–Meier and Cox Proportional Hazards Regression Analyses

To further support the robustness of our results, we performed Cox proportional hazards regression analysis. The risk of all‐cause death (HR: 1.96, 95% CI: 1.06–3.63, *p* = 0.03) and MACCEs (HR: 2.63, 95% CI: 1.73–4.02, *p* < 0.0001) were statistically significantly higher in the moderate/severe calcification group than in the none/mild calcification group. Additionally, in patients with calcified nodules, Cox regression analysis demonstrated a statistically significantly increased risk of all‐cause death (hazard ratio [HR]: 2.88, 95% CI: 1.21–6.86, *p* = 0.03) and MACCE (HR: 4.78, 95% CI: 2.91–7.86, *p* < 0.0001). Regarding post‐PCI ACEI use, there was no significant difference in MACCE incidence in the none/mild calcification group (HR, 0.94; 95% CI: 0.39–2.29, *p* = 0.90). In contrast, in the moderate/severe calcification group, ACEI use was associated with a statistically significantly lower incidence of MACCEs (HR: 0.26, 95% CI: 0.07–0.90, *p* = 0.02). These consistent results from both Kaplan–Meier and Cox regression analyses support the statistical significance and reliability of our findings.

## 4 Discussion

In this study, we investigated factors associated with coronary artery calcification, including calcified nodules, and their clinical outcomes in target lesions. The main findings of this study were as follows: First, patients with moderate/severe coronary calcification in culprit lesions had higher incidences of both all‐cause death and MACCEs than those with none/mild calcification. Second, the rate of moderate/severe coronary calcification in culprit lesions was significantly lower in patients using ACEI than in those using ARB. Third, CKD and β‐blocker use were positively associated with both moderate/severe calcification and calcified nodules, whereas ACEI use was inversely associated with both moderate/severe calcification and calcified nodules in culprit lesions for PCI. Finally, among patients with moderate/severe calcification, the incidence of MACCEs after PCI was significantly lower in patients with ACEI use than in those without ACEI use.

We conducted a logistic regression analysis of the factors influencing moderate/severe calcification of the target lesion and calcified nodules in the target lesions as a sensitivity analysis, without adjustment for age, to examine its potential impact on the associations (Supporting Information S1: Tables S1 and S2). The results demonstrate that while age is a significant factor, the overall trends in the associations remain consistent, further supporting our conclusions.

Coronary artery calcification is a poor prognostic factor in patients with CAD [9, 28, 29]. Additionally, PCI for lesions with severe calcification or calcified nodules has not yielded satisfactory results [8, 9, 11, 12]. The results of this study also support those of previous reports. However, this study newly suggests that the use of ACEI has a post‐PCI event‐preventive effect when the target lesion assessed using IVUS during PCI has moderate or severe calcification or is a stenotic lesion with calcified nodules.

The presence of severe calcification in coronary artery lesions is a major obstacle to interventional treatment. In coronary artery interventions, it is important to appropriately dilate the stenotic site and avoid malapposition during stent placement [30]. Therefore, intracoronary imaging‐guided PCI is useful for evaluating procedural and long‐term clinical outcomes [31]. However, in severely calcified stenosis, balloon dilation cannot sufficiently expand the vascular lumen, and some form of lesion modification, such as RA or OAS, is required [12, 21, 32]. Furthermore, despite these treatments, PCI for calcified nodules remains a major problem that does not ensure adequate outcomes [33, 34].

ACEIs have been shown to reduce morbidity and mortality in patients with cardiovascular diseases, including heart failure and left ventricular systolic dysfunction [17, 18, 35]. In addition to the established benefits in these populations, previous studies have confirmed that ACEIs reduce major cardiovascular events and all‐cause mortality in high‐risk patients with atherosclerosis and diabetes [36].

The renin‐angiotensin system (RAS) is an important regulator of cardiovascular disease and plays an important role in vascular pathophysiology [37]. Angiotensin (Ang) II, which is produced from Ang I as a product of ACE, is the major bioactive peptide in the RAS. Ang II leads to vasocontractile, inflammatory, and oxidative actions by activating AT1 receptors. ACEIs are a class of medications used to treat and manage hypertension, a significant risk factor for coronary disease, heart failure, stroke, and other cardiovascular conditions. ACEIs prevent the production of Ang Ⅱ, a substance that narrows blood vessels, thereby lowering blood pressure. ARBs directly inhibit the binding of Ang II to Ang II type 1 receptors.

The components of the renin‐angiotensin‐aldosterone system (RAAS), such as renin, angiotensinogen, and ACE, are expressed in various tissues throughout the body. The produced Ang II is associated with inflammation and immune reactions, such as autocrine and paracrine processes, including tissue fibrosis and cell hypertrophy, leading to organ damage. Therefore, ACEIs are commonly indicated for conditions such as heart failure, acute coronary syndrome, nephrotic syndrome, diabetes, and hypertension [38]. Furthermore, immunohistological examination of coronary artery atherosclerotic lesions has shown that ACE is expressed in macrophages of highly active coronary artery atherosclerotic lesions, produces Ang II, and increases oxidative stress in macrophages [39]. This is believed to contribute to the progression of arteriosclerosis.

ACEIs and ARBs affect the RAS. ACEI treatment can enhance total nitric oxide (NO) concentration and modulate NO‐dependent endothelial function because it affects the bradykinin receptor system [40]; however, ARB does not affect the bradykinin receptor system. NO plays a significant role in vascular calcification. A previous report revealed that NO inhibits vascular smooth muscle cell calcification and osteoblastic differentiation, preventing calcification in vascular grafts [41]. Thus, NO may have a protective effect against vascular calcification, which is associated with cardiovascular events and increased mortality.

Furthermore, NO modulates the release of various inflammatory mediators, acts as an antioxidant, inhibits apoptosis, and promotes cellular survival [42].

Inflammation and calcification play important roles in the progression of atherosclerosis. Inflammation is an important process that promotes plaque progression and calcification. Repeated inflammation causes the development of atherosclerosis and calcification of vessel walls [43]. Therefore, anti‐inflammatory effects and regulation of the immune system and cell apoptosis are also involved in vascular calcification, and the role of NO has been considered. The heptapeptide Ang‐(1‐7) has been attracting attention as a factor involved in suppressing cardiovascular fibrosis, inflammation, antioxidation, and calcification [44]. Ang‐(1‐7) plays a major role in the protective branch of the RAS. It primarily binds to the G protein‐coupled receptor Mas and counters the actions of Ang II, which increases calcium influx and cell adhesion and promotes cytokine release [45]. Ang‐(1‐7) is produced from Ang II through the action of ACE2 and from Ang I via neutral endopeptidase activity. ACEIs affect the Ang‐(1‐7) pathway by increasing Ang‐(1‐7) levels. Ang‐(1‐7) is an efficient inhibitor of fibrosis and calcification within the vasculature by counteracting the deleterious effects of Ang II [46].

Furthermore, Ang‐(1‐7) attenuates endothelial dysfunction and exhibits antiproliferative, anti‐fibrotic, and anti‐inflammatory effects, all of which contribute to the prevention of vascular calcification [45]. Moreover, long‐term treatment with ARB increases Ang II levels through a positive feedback loop, increasing the expression of plasminogen activator inhibitor‐1, which causes inflammation and fibrosis. Ang II also enhances the activity of matrix metalloproteinase‐2 and metalloproteinase‐9 and causes the degradation of elastin, which has calcium‐binding capacity and may induce calcium deposition in tissues and support the mineralization process [47]. In contrast, ACEIs reduce Ang II levels by blocking ACEs. β‐Blocker use was significantly associated with moderate/severe calcification in this study. β‐Blockers have a dual role as follows: antihypertensive and cardiovascular protective effects in patients with hypertension. They are also important drugs in ischemic heart disease. However, we observed that β‐blocker use was an independent risk factor for moderate/severe calcification in the target lesion for PCI. Animal and cell biology studies have demonstrated that β‐blockers can have profibrotic effects by activating the production of transforming growth factor‐β and collagen I and III [48]. Furthermore, β‐blockers worsen insulin sensitivity, alter lipid metabolism, and cause weight gain [49]. Disorders in insulin sensitivity and lipid metabolism result in endothelial dysfunction. β‐Blocker use is also associated with progressive calcification. Understanding specific mechanisms and clinical implications of β‐blockers on vascular calcification requires further investigation.

CAD is significantly more prevalent in patients with CKD, and vascular calcification is a key pathological feature that supports the findings of our study [50]. The severity of calcification in culprit coronary lesions tends to increase with CKD progression [51]. The mechanisms underlying vascular calcification are complex and multifactorial, and the precise causes of CAC remain unclear. For the management of CAD, regression of atherosclerotic plaques is essential to prevent future cardiovascular events, and statins and RAAS inhibitors serve as cornerstone therapies.

Our findings suggest that ACE inhibitors may help suppress the formation of coronary artery calcification (CAC) and potentially reduce cardiovascular events following PCI for calcified lesions. Given their established use in patients with hypertension and atherosclerotic disease, ACEIs could be considered a therapeutic option in patients with calcified coronary lesions. However, these results remain speculative, and further large‐scale prospective studies using advanced imaging modalities are required to validate their role in optimizing PCI outcomes.

These findings are consistent with prior reports suggesting the involvement of ACEIs in modulating inflammation, oxidative stress, and endothelial function, all of which contribute to the progression of vascular calcification. However, our study is among the first to evaluate the association between ACEI use and IVUS‐assessed calcification severity, as well as clinical outcomes following PCI in patients with calcified coronary lesions. This adds a new perspective to the current body of evidence and may suggest an additional therapeutic role for ACEIs in managing calcified coronary artery disease.

In clinical practice, coronary calcification remains a major challenge in PCI, as it is associated with suboptimal procedural results and worse long‐term outcomes. The potential use of ACEIs in this context may offer an additional therapeutic strategy, particularly in patients with stable angina and calcified stenosis undergoing PCI.

This study has some limitations. First, it was a retrospective study conducted in a small cohort of patients at a single institution. In addition, this study was conducted at a single center in Japan, which may limit the generalizability of our findings to other populations with different demographic and clinical characteristics. Future studies should involve larger multicenter cohorts and prospective designs to validate our findings across diverse populations. Second, CAC in the target lesion after PCI was assessed using IVUS. IVUS is operator‐dependent and subject to interobserver variability. Although core laboratory analysis was not performed in this study, IVUS data were independently reviewed by multiple PCI specialists who strictly followed the standardized criteria for calcification assessment to minimize measurement error. Nevertheless, future studies should consider incorporating core laboratory analyses to enhance reproducibility and objectivity. Previous studies have frequently evaluated coronary calcification using multislice CT or fluoroscopy [52, 53]; however, multislice CT could not be performed in all patients. A combination of IVUS and CT‐based modalities may allow for more comprehensive evaluation of calcification in future research. Third, we could not completely exclude systemic illnesses, such as occult malignancies or other frailty‐affecting outcomes. Further research, including broader clinical information, such as frailty indices or cancer screening data, may help clarify these confounding factors. Fourth, as the PCI procedure was left to the discretion of the operator, there was no standardized strategy regarding the devices used, including the DES, DCB, and debulking system. Future prospective studies with standardized procedural protocols are needed to determine the optimal intervention strategy, particularly in patients with severe calcifications. Lastly, except for C‐reactive protein, other markers of inflammation that may have influenced the occurrence of moderate/severe calcification were not examined. The inclusion of additional inflammatory markers (e.g., interleukins, tumor necrosis factor‐α) in future studies may provide deeper insights into the relationship between inflammation and calcification. To address these limitations, we plan to use multi‐imaging modalities and further sample data in future studies to verify our results.

In conclusion, IVUS‐assessed moderate/severe calcification is a significant predictor of all‐cause death and MACCE in patients undergoing PCI. ACEI use was negatively associated with the presence of calcification and calcified nodules, and may reduce the incidence of MACCE in patients with calcified lesions. These findings highlight the importance of calcification assessment in risk stratification and suggest a potential role of ACEIs in modulating calcification. To confirm these associations and clarify causality, a prospective randomized controlled trial using multi‐modality imaging is warranted.

## Author Contributions

**Daisuke Kanda:** conceptualization, data curation, formal analysis, investigation, methodology, visualization, writing – original draft. **Akihiro Tokushige:** formal analysis, methodology, writing – review and editing. **Kenta Ohmure:** data curation. **Daichi Fukumoto:** data curation. **Hirokazu Shimono:** data curation. **Hiroyuki Tabata:** data curation. **Nobuhiro Ito:** data curation. **Takuro Kubozono:** writing – review and editing. **Mitsuru Ohishi:** conceptualization and supervision.

## Conflicts of Interest

The authors declare no conflicts of interest.

## Transparency Statement

The lead author, Daisuke Kanda, affirms that this manuscript is an honest, accurate, and transparent account of the study being reported, that no important aspects of the study have been omitted, and that any discrepancies from the study as planned (and, if relevant, registered) have been explained.

## Supporting information

Supplementary Table S1_S2_clean.

## Data Availability

Daisuke Kanda had full access to all of the data in this study and takes complete responsibility for the integrity of the data and the accuracy of the data analysis. The data sets used and/or analyzed during the current study are available from the corresponding author upon reasonable request.
