# Supplementary material for: Impact of Angiotensin‐Converting Enzyme Inhibitor on Coronary Artery Calcification Evaluated by Intravascular Ultrasound: A Retrospective Cohort Study
Source: Health Sci Rep. 2025 Jun 11;8(6):e70900. doi: 10.1002/hsr2.70900 (PMC12158666; doi:10.1002/hsr2.70900)
Supplement: Supplementary file 1 — Supplementary Table S1_S2_clean. [file HSR2-8-e70900-s001.docx]

**Table S1.** Logistic regression analysis of factors influencing moderate/severe calcification of the target lesion as a sensitivity analysis without adjustment for age

|  | Univariate analysis | | |  | Multivariate analysis | | |
| --- | --- | --- | --- | --- | --- | --- | --- |
|  | OR | (95% CI) | p-value |  | OR | (95% CI) | p-value |
| Age | 1.03 | (1.02–1.05) | <0.001 |  |  |  |  |
| Sex (male) | 1.06 | (0.74–1.52) | 0.75 |  |  |  |  |
| BMI | 0.96 | (1.00–1.04) | 0.06 |  |  |  |  |
| Hypertension | 1.40 | (0.87–2.23) | 0.17 |  |  |  |  |
| Diabetes mellitus | 1.23 | (0.89–1.71) | 0.21 |  |  |  |  |
| Dyslipidemia | 0.80 | (0.55–1.15) | 0.22 |  |  |  |  |
| Current smoking | 0.67 | (0.44–1.01) | 0.06 |  |  |  |  |
| CKD | 2.48 | (1.78–3.47) | <0.001 |  | 2.39 | (1.71–3.36) | <0.001 |
| Calcium channel blocker use | 0.99 | (0.72–1.37) | 0.98 |  |  |  |  |
| ACEI use | 0.41 | (0.22–0.72) | 0.003 |  | 0.56 | (0.36–0.89) | 0.01 |
| ARB use | 1.16 | (0.84–1.59) | 0.37 |  |  |  |  |
| β-Blocker use | 1.65 | (1.19–2.28) | 0.003 |  | 1.68 | (1.20–2.37) | 0.003 |
| Statin use | 1.20 | (0.80–1.80) | 0.37 |  |  |  |  |
| hs-CRP ≥0.1 mg/dL | 0.88 | (0.64–1.22) | 0.45 |  |  |  |  |
| LDL-C >70 mg/dL | 0.75 | (0.54–1.03) | 0.07 |  |  |  |  |
| HDL-C <40 mg/dL | 0.74 | (0.49–1.10) | 0.13 |  |  |  |  |
| Albumin <3.5 g/dL | 1.49 | (0.96–2.33) | 0.08 |  |  |  |  |
| FPG | 0.998 | (0.994–1.002) | 0.39 |  |  |  |  |

ACEI, angiotensin-converting enzyme inhibitor; ARB, angiotensin II receptor blocker; BMI, body mass index; CI, confidence interval; CKD, chronic kidney disease; FPG, fasting plasma glucose; HDL-C, high-density lipoprotein cholesterol; hs-CRP, high-sensitivity C-reactive protein; LDL-C, low-density lipoprotein cholesterol; OR, odds ratio

**Table S2.** Logistic regression analysis of factors influencing calcified nodules in the target lesions as a sensitivity analysis without adjustment for age

|  | Univariate analysis | | |  | Multivariate analysis | | |
| --- | --- | --- | --- | --- | --- | --- | --- |
|  | OR | (95% CI) | p-value |  | OR | (95% CI) | p-value |
| Age | 1.00 | (0.97–1.03) | 0.96 |  |  |  |  |
| Sex (male) | 2.12 | (0.87–5.14) | 0.09 |  |  |  |  |
| BMI | 0.95 | (0.87–1.03) | 0.22 |  |  |  |  |
| Hypertension | 7.03 | (0.95–51.77) | 0.06 |  |  |  |  |
| Diabetes mellitus | 1.04 | (0.55–2.00) | 0.85 |  |  |  |  |
| Dyslipidemia | 1.01 | (0.48–2.12) | 0.97 |  |  |  |  |
| Current smoking | 0.68 | (0.28–1.65) | 0.38 |  |  |  |  |
| CKD | 3.42 | (1.55–7.53) | 0.002 |  | 3.16 | (1.43–7.01) | 0.005 |
| Calcium channel blocker use | 1.32 | (0.70–2.49) | 0.97 |  |  |  |  |
| ACEI use | 0.11 | (0.01–0.81) | 0.03 |  | 0.10 | (0.01–0.74) | 0.02 |
| ARB use | 1.67 | (0.88–3.17) | 0.11 |  |  |  |  |
| β-Blocker use | 1.93 | (1.02–3.64) | 0.04 |  | 2.06 | (1.08–3.93) | 0.02 |
| Statin use | 3.31 | (1.004–10.00) | 0.49 |  |  |  |  |
| hs-CRP ≥0.1 mg/dL | 1.41 | (0.72–2.75) | 0.30 |  |  |  |  |
| LDL-C ≥70 mg/dL | 0.69 | (0.36–1.31) | 0.25 |  |  |  |  |
| HDL-C <40 mg/dL | 1.38 | (0.67–2.84) | 0.37 |  |  |  |  |
| Albumin < 3.5 g/dL | 1.44 | (0.64–3.23) | 0.37 |  |  |  |  |
| FPG | 0.997 | (0.989–1.006) | 0.55 |  |  |  |  |

ACEI, angiotensin-converting enzyme inhibitor; ARB, angiotensin II receptor blocker; BMI, body mass index; CI, confidence interval; CKD, chronic kidney disease; FPG, fasting plasma glucose; HDL-C, high-density lipoprotein cholesterol; hs-CRP, high-sensitivity C-reactive protein; LDL-C, low-density lipoprotein cholesterol; OR, odds ratio
